# The effect of abaloparatide on the proximal femur in men with osteoporosis assessed by three-dimensional dual-energy X-ray absorptiometry

**DOI:** 10.1093/jbmrpl/ziaf098

**Published:** 2025-06-05

**Authors:** Ruban Dhaliwal, John Boxberger, Yamei Wang, Bruce H Mitlak, Ludovic Humbert, Neil Binkley

**Affiliations:** Division of Endocrinology, Department of Internal Medicine, The University of Texas Southwestern Medical Center, Dallas, TX 75390, United States; Endocrine Unit, Massachusetts General Hospital, Boston, MA 02114, United States; Business and Corporate Development, Radius Health Inc., Boston, MA 00210, United States; Biostatistics, Radius Health Inc., Boston, MA 00210, United States; Clinical Development, Radius Health Inc., Boston, MA 00210, United States; President and Scientific Development, 3D-Shaper Medical, Barcelona 08007, Spain; Osteoporosis Clinical Research Program, University of Wisconsin-Madison, Madison, WI 53705, United States

**Keywords:** abaloparatide, cortical volumetric BMD, osteoporosis, 3D modeling, DXA

## Abstract

Abaloparatide treatment significantly increased BMD at the LS, TH, and FN compared with placebo in men with osteoporosis in the phase 3 ATOM trial. The current study used 3D-DXA modeling to evaluate the effects of abaloparatide on cortical and trabecular compartments of the proximal femur in ATOM study participants. Proximal femur DXA images were retrospectively analyzed using 3D-DXA (3D-Shaper software v2.12.0, 3D-Shaper Medical, Barcelona, Spain) to evaluate changes in bone parameters from baseline at months 6 and 12 in all randomized men from the ATOM trial. Between-group comparisons were made for percent change from baseline data based on a mixed-effect repeated-measure model with treatment, visit, treatment-by-visit interaction, and type of DXA scanner as fixed effects. Other covariates include BMI, age, and baseline values of bone parameters. Abaloparatide treatment significantly increased integral volumetric BMD (vBMD) (3.7%), trabecular vBMD (7.0%), cortical thickness (1.1%), and cortical surface BMD (1.7%) at 12 mo compared to baseline (*p* < .0001). Changes were greater for abaloparatide compared to placebo for all 4 parameters (*p* < .01). Significant increases from baseline compared to placebo in integral vBMD (2.7% vs −0.1%, *p* < .0001) and trabecular vBMD (6.1% vs −0.6%, *p* < .0001) were also observed at 6 mo. In conclusion, in men with osteoporosis, abaloparatide improved proximal femur 3D-DXA parameters broadly consistent with results in postmenopausal women in the ACTIVE study, adding to the growing data on abaloparatide bone structure effects at the hip.

## Graphical Abstract

**Graphical Abstract:**
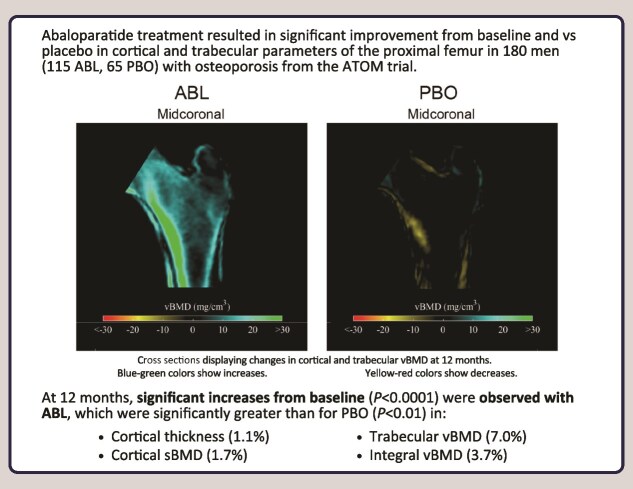

## Introduction

Osteoporosis is a growing public health burden given population aging.^1^ Osteoporotic fractures are associated with increased mortality, disability, and higher rates of hospitalization, with hip fractures carrying a particularly high burden.^1^^,^^2^ Changes in both trabecular and cortical bone occur during aging that adversely impact bone strength and fracture risk.^3^^,^^4^

The proximal femur is a complex structure that includes the FN, trochanter, and proximal femoral shaft subregions.^5^ Each of these subregions differs in its cortical versus trabecular composition and in biomechanical loading.^6^ In older US populations, hip fractures have been reported to occur in equal proportions at the FN and trochanteric regions,^7^ which are considered mixed sites with both cortical and trabecular components.^8^ Evaluating BMD changes in the different compartments within the hip and the impacts of these changes on biomechanical strength can aid in the evaluation of fracture risk in patients with osteoporosis.^9–11^

DXA is a well-established method to measure areal BMD (aBMD) in vivo and correlates well with biomechanically determined bone strength.^12^ Three dimensional (3D)-DXA modeling has been used to process 2D hip DXA scans to provide patient-specific QCT-like data and to better understand the mechanisms by which osteoporosis therapies affect cortical and trabecular bone mass.^10^^,^^13–16^ The majority of these data are derived from clinical studies in women, with only one of the studies, including a small proportion of men. The accuracy of 3D-DXA cortical and trabecular parameters has been validated in men and women with variable BMD (normal, osteopenia, and osteoporosis), and a high correlation between 3D-DXA and QCT-derived measurements shown.^17^ Specifically, correlation coefficients between 3D-DXA and QCT were 0.86, 0.93, and 0.95 for vBMD at trabecular, cortical, and integral compartments, respectively.

In the Abaloparatide Comparator Trial In Vertebral Endpoints (ACTIVE) study, 18 mo of treatment with abaloparatide, a selective PTH receptor type 1 agonist, increased aBMD and reduced the risk of vertebral, nonvertebral, clinical, and major osteoporotic fractures compared to placebo in postmenopausal women with osteoporosis.^18^^,^^19^ Abaloparatide treatment produced significant improvements in trabecular volumetric BMD (Tb.vBMD) and cortical vBMD (Ct.vBMD) at all 3 hip subregions as assessed by 3D-DXA.^20^ Abaloparatide has also been shown to increase BMD in men with osteoporosis^21^; however, the effects of abaloparatide treatment on trabecular and cortical regions in men have not been previously studied. The current 3D-DXA modeling analysis evaluates the effects of 6 and 12 mo of abaloparatide treatment on cortical and trabecular compartments of the proximal femur in men with osteoporosis from the ATOM trial.

## Materials and methods

### Study design

The ATOM trial was a randomized, double-blind, phase 3 study evaluating the efficacy and safety of 12 mo of treatment with abaloparatide (80 μg) compared with placebo in men with primary osteoporosis or osteoporosis associated with hypogonadism and has been previously described.^21^ Briefly, men aged 40-85 yr with BMD T-scores ≤−2.5 and >−3.5 at the LS, TH, or FN or ≤−1.5 with a history of radiologic vertebral fracture or low trauma nonvertebral fracture within 5 yr preceding the study were included. Men older than 65 yr who did not meet fracture criteria with a T-score of ≤−2.0 were also eligible for study inclusion. Men with osteoporosis associated with hypogonadism were required to be on a stable dose of androgen replacement therapy for at least 12 mo. Potential study participants were excluded, if they had any prior treatment with PTH or PTHrP-derived therapies or intravenous bisphosphonates, if they had been treated with oral bisphosphonates within the past 3 yr, or treated with denosumab within the past 18 mo. Men with conditions or taking medication associated with secondary osteoporosis were also excluded, including history of Cushing disease, growth hormone deficiency or excess, hyperthyroidism, hypo- or hyperparathyroidism or malabsorptive syndromes within the past year, treatment with anticonvulsants that affect vitamin D metabolism, treatment with anabolic steroids within 90 d, and daily treatment with oral, intranasal, or inhaled corticosteroids within 12 mo.

**Table 1:** Baseline demographics and clinical characteristics.

	Abaloparatide (*n* = 115)	Placebo (*n* = 65)	Overall (*n* = 180)
**Age (yr)**			
**Mean (SD)**	67.9 (8.1)	67.6 (8.4)	67.8 (8.2)
**Median (min, max)**	68 (44, 84)	69 (42, 82)	69 (42, 84)
**BMI (kg/m^2^), mean (SD)**	26.5 (3.5)	26.2 (3.4)	26.4 (3.5)
**aBMD T-score, mean (SD)**			
**LS**	−2.1 (1.1)	−2.1 (1.1)	−2.1 (1.1)
**TH**	−1.6 (0.6)	−1.9 (0.7)	−1.7 (0.7)
**FN**	−2.1 (0.6)	−2.3 (0.6)	−2.2 (0.6)
**Baseline prevalent vertebral fracture, *n* (%)**	42 (36.5)	23 (35.4)	65 (36.1)
**No prior fracture, *n* (%)**	47 (40.9)	25 (38.5)	72 (40.0)
**Primary hypogonadism**	4 (3.5)	0	4 (2.2)
**Secondary hypogonadism**	1 (0.9)	0	1 (0.6)

### 3D-DXA analysis

In this retrospective exploratory analysis, hip DXA images from randomized participants in the ATOM trial with DXA scans at baseline, month 6, and month 12 were assessed. The left hip, where possible, was used for all DXA analyses, with the right side used in men where the left was not amenable to scanning (ie, left hip implant). Blinded image files were analyzed by 3D-Shaper Medical using 3D-Shaper software (v2.12.0, 3D-Shaper Medical, Barcelona, Spain) as previously described.^14^ Changes from baseline at months 6 and 12 were assessed at the proximal femur for Ct.vBMD, cortical thickness (Ct.Th), cortical surface BMD (Ct.sBMD), Tb.vBMD, and integral vBMD (Int.vBMD).

The 3D data generated from the hip DXA scans were used to assess the anatomical distribution of bone structure in each group. An average 3D model was generated per group and time point using image registration techniques. The average 3D models for each group obtained at the follow-up time points were compared to their respective baseline models to assess anatomical distribution of changes in bone structure. Changes in Ct.Th, vBMD, and sBMD were displayed at the periosteal surface of the femur using 3D visualizations. Changes in cortical and trabecular vBMD were displayed in the midcoronal, neck, intertrochanteric, and lower shaft cross sections.

### Statistical analysis

All statistical analyses were performed using the Statistical Analysis System ([SAS] software version 9.4, SAS Institute Inc., Cary, NC, USA). Participants in the ATOM trial that had TH DXA images at baseline, month 6, and month 12 were included in the 3D-DXA analysis. Within-group comparisons for change from baseline at months 6 and 12 were made using paired *t* tests. Between-group comparisons for percent change from baseline were made using a mixed-effect repeated-measure model with treatment, visit, treatment-by-visit interaction, and type of DXA scanner as fixed effects. Covariates included BMI, age, and bone parameter values at baseline. *p* values <.05 were considered significant.

### Ethics

The ATOM study from which the DXA scans were procured was conducted in accordance with the International Conference on Harmonization, the Declaration of Helsinki (2013), and applicable local regulations. Local institutional or central internal review boards (IRBs; for some countries) were used to obtain approval from all institutions. All participants provided informed written consent to participate in the study.

## Results

Of the participants in the ATOM trial, 180 (115 from the abaloparatide group, 65 from the placebo group) had a hip DXA image at baseline, month 6, and month 12 and were included in the 3D-DXA analysis. The baseline demographics and clinical characteristics (Table 1) were similar between groups including age, baseline aBMD, and prior fracture history.

**Figure 1:**
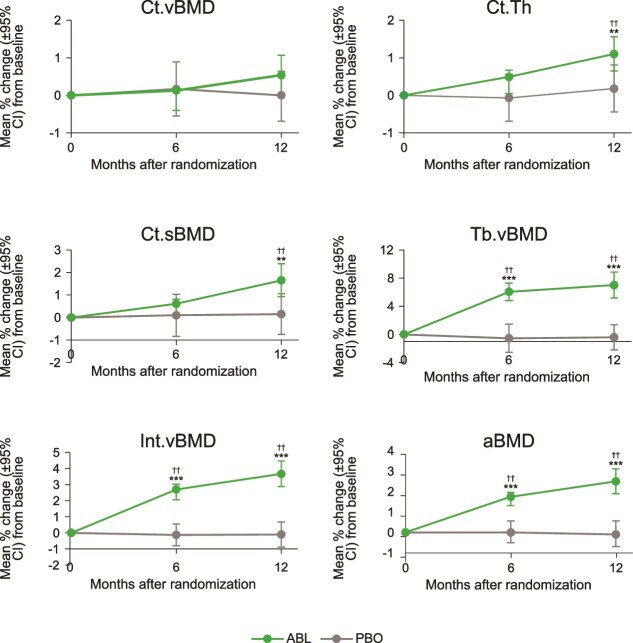
Mean percent change in DXA and 3D-DXA parameters at the proximal femur from baseline to 12 mo. Abbreviations: 3D-DXA, three-dimensional DXA; ABL, abaloparatide; aBMD, areal BMD; Ct.sBMD, cortical sBMD; Ct.Th, cortical thickness; Ct.vBMD, cortical vBMD; Int.vBMD, integral vBMD; PBO, placebo; sBMD, surface BMD; Tb.vBMD, trabecular vBMD; vBMD, volumetric BMD. Longitudinal changes in hip DXA (aBMD) and 3D-DXA endpoints at 0, 6, and 12 mo after randomization. Data shown as mean ± 95% CI for percent change from baseline. ^*^^*^p < .01 vs PBO. ^*^^*^^*^p < .0001 vs PBO. ^††^*p* < .0001 vs baseline.

Data are presented as mean percent change from baseline ± SD unless otherwise stated. After 12 mo of abaloparatide treatment, significant within-group percent increases from baseline were observed in Ct.Th (1.1% ± 2.5), Ct.sBMD (1.7% ± 3.9), Tb.vBMD (7.0% ± 10.0), and Int.vBMD (3.7% ± 4.3), all *p* < .001. Mean percent change from baseline for all 4 variables (Ct.Th, Ct.sBMD, Tb.vBMD, and Int.vBMD) were greater for men treated with abaloparatide compared with men treated with placebo (*p* < .01) at 12 mo (Figure 1). Significant increases from baseline in Tb.vBMD (6.1% ± 6.7), Int.vBMD (2.7% ± 3.5), and Ct.Th (0.5% ± 2.4) were seen in men treated with abaloparatide at 6 mo (*p* < .05), and these changes were significant compared to placebo for Tb.vBMD (least square mean [LSM] difference 7.3 [95% CI 5.1-9.5]; *p* < .0001) and Int.vBMD (LSM difference 3.0 [95% CI 2.0-4.0]; *p* < .0001). A significant increase from baseline in aBMD was also observed in the abaloparatide group compared to placebo (LSM difference 2.0 [95% CI 1.3-2.7] at 6 mo, *p* < .0001; 2.8 [95% CI 1.9-3.8] at 12 mo, *p* < .0001). Changes in DXA and 3D-DXA parameters by hip DXA subregion (ie, FN, trochanter, shaft) are shown in Table S1.

**Figure 2:**
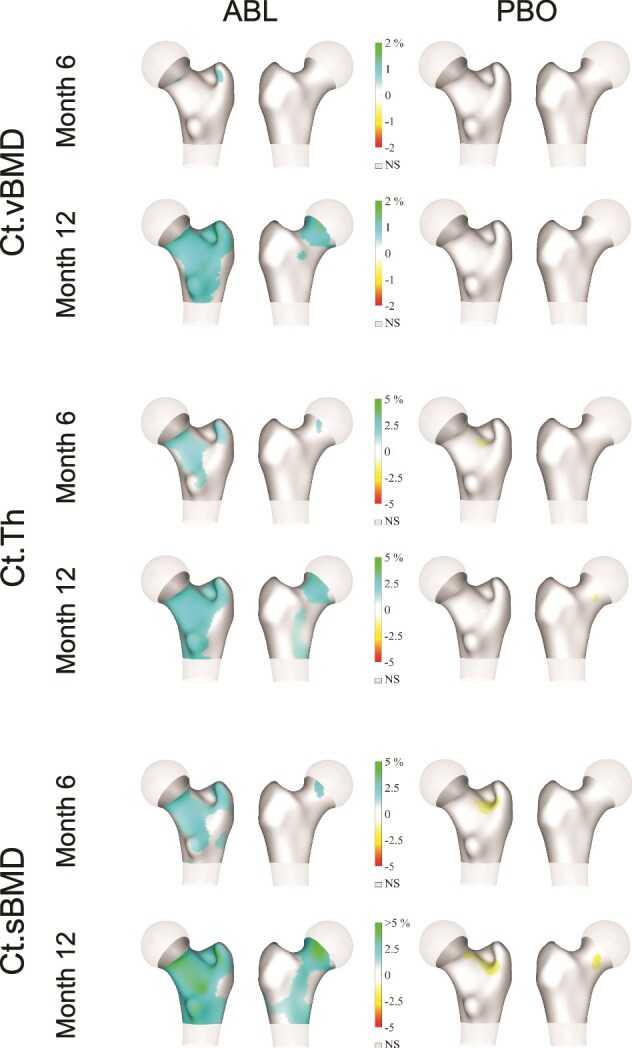
3D visualizations of the percent change from baseline in cortical vBMD, thickness, and sBMD. Abbreviations: ABL, abaloparatide; Ct.sBMD, cortical sBMD; Ct.Th, cortical thickness; Ct.vBMD, cortical vBMD; NS, not significant; PBO, placebo; sBMD, surface BMD; vBMD, volumetric BMD. Average spatial changes in 3D-DXA cortical endpoints at months 6 and 12. For each endpoint and treatment, anterior (left) and posterior (right) views of a standardized proximal femur model are shown. Blue-green colors represent increases and yellow-red colors represent decreases. NS: not significant against baseline.

The distribution of the changes in Ct.vBMD, Ct.Th, and Ct.sBMD at the periosteal surface of the femur after 6 and 12 mo of treatment with abaloparatide or placebo is shown in Figure 2. At 12 mo, increases of >1% in Ct.vBMD (predominantly at the FN), >2.5% in Ct.Th, and >5% in Ct.sBMD (both at the FN, trochanter, and shaft at posterior and medial aspects) were observed in the group treated with abaloparatide compared to no change or decreases in the placebo group in all categories measured.

**Figure 3:**
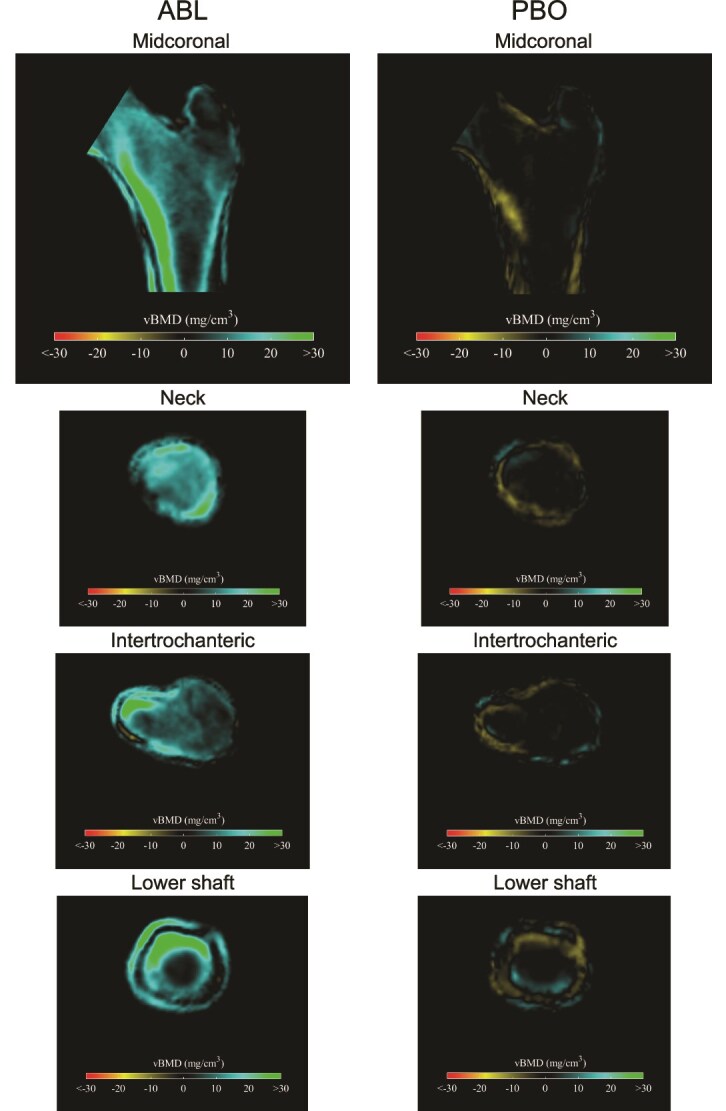
2D cross-sectional changes in cortical and trabecular vBMD at month 12. Abbreviations: ABL, abaloparatide; PBO, placebo; vBMD, volumetric BMD. Cross sections displaying changes in cortical and trabecular vBMD at 12 mo in the ABL and PBO groups. Increases are presented in blue-green colors and decreases are presented in yellow-red colors.

Cross-sectional images generated using 3D-Shaper to display changes in cortical and trabecular vBMD at 12 mo in the abaloparatide-treated and placebo-treated groups are shown in Figure 3. Increases are presented in blue-green colors and decreases are presented in yellow-red colors. Increases in vBMD were observed in the midcoronal, neck, intertrochanteric, and lower shaft in the group treated with abaloparatide compared to losses in all areas in the placebo group.

## Discussion

Hip fractures represent a global burden to individuals and healthcare systems, with potentially greater impacts on morbidity, mortality, and loss of independence reported in men compared with women.^22^ This retrospective analysis utilized 3D-DXA imaging to evaluate the effects of abaloparatide on trabecular and cortical compartments of the proximal femur. After 6 mo, trabecular 3D-DXA parameters at the proximal femur were significantly improved in men with osteoporosis treated with abaloparatide compared with placebo. After 12 mo of abaloparatide treatment, significant increases in cortical sBMD and trabecular vBMD were observed. Increases in the trabecular compartment at the femur neck appear higher than in the shaft and trochanteric compartment with abaloparatide (Figure 3; blue shading is more prominent in frontal slice). The findings in the FN may prove clinically beneficial, as FN fractures are among the most common type of hip fractures in both men and women.^23^^,^^24^ In men, hip fractures are associated with greater morbidity/mortality compared with women.^25^^,^^26^ Given that BMD is a key determinant of fracture risk, these site-specific BMD gains may translate to reduction in fracture incidence.^27^ The progressive compartmental efficacy demonstrated through 12 mo, with significant changes in cortical bone occurring later in the time-course, highlights the continued effects of abaloparatide over time and emphasizes the need for adherence and persistence to maximize the therapeutic benefit in men with osteoporosis.

The findings of trabecular vBMD gain with abaloparatide are consistent with significant 12-mo gains in LS aBMD, a predominantly trabecular anatomic site, previously reported from the ATOM study.^21^ The findings in this report are consistent with those in women from the ACTIVE study, which demonstrated significant improvements in cortical and trabecular compartment 3D-DXA parameters of the proximal femur.^19^^,^^20^ Likewise, an exploratory analysis in Japanese patients at high fracture risk enrolled in the ACTIVE-J trial (which pooled data from 186 women and 20 men) showed an increase in vBMD in the total and trabecular region of the FN with abaloparatide as assessed by QCT.^28^

Preclinical studies have demonstrated that abaloparatide increases bone mass and density at both trabecular and cortical sites.^29^ Normal skeletal aging in men is associated with loss of trabecular thickness and reductions in cortical BMD, with increased trabecularization of the cortex and periosteal apposition.^11^^,^^30–32^ It is possible that abaloparatide treatment in men with osteoporosis at high risk for fracture may enhance trabecular bone formation, increase periosteal and reduce endocortical apposition, or lead to greater filling of new remodeling sites, all of which are important predictors of bone strength/fragility.^33–35^

Few studies have evaluated the compartmental effects of osteoporosis treatments in men. However, in men with glucocorticoid-induced osteoporosis, HRQCT analysis measured at the spine showed that 18 mo of teriparatide treatment significantly increased integral and trabecular aBMD compared to risedronate, while changes in cortical BMD and Ct.Th were not different between the 2 treatment groups.^36^ These findings highlight differences in response to treatment in the cortical and trabecular compartments for antiresorptive and anabolic therapies in men as well as potential differences between abaloparatide and teriparatide with regard to Ct.Th, although additional studies are needed in this regard. While HR-pQCT is currently utilized primarily in research settings, it may improve fragility fracture prediction beyond that of DXA and commonly used algorithms.^37^ Other studies have utilized HR-pQCT to determine impacts of different therapies on cortical and trabecular bone microstructure, highlighting the potential uses for these analyses, including in fracture prediction tools or as endpoints in clinical trials.^38^ The use of HR-pQCT is limited due to its higher cost and radiation dose compared to other imaging techniques. In contrast, 3D-DXA imaging offers cortical and trabecular parameters using DXA, a standard modality in both clinical practice and research settings, showing great potential for providing deeper insights into the effects of pharmacological treatments on bone health.

Limitations of this study include those associated with the 3D-DXA modeling approach, as described previously.^13^ The software for 3D-DXA modeling was developed to mimic QCT based on 2D images from a treatment-naïve population, and the effect of osteoporosis treatment on this relationship is unknown.^17^ In addition, the patient population in ATOM was limited to men aged 40-85 yr with primary osteoporosis or osteoporosis associated with hypogonadism and extrapolation of the results to a broader population cannot be done.^21^ Additionally, the results were consistent with what was seen in postmenopausal women at high risk for fracture who were treated with abaloparatide in the ACTIVE trial and in post hoc analyses of ACTIVE using 3D-DXA modeling.^14^^,^^19^^,^^20^

In conclusion, 12 mo of treatment with abaloparatide improved both trabecular and cortical 3D-DXA parameters at the proximal femur in men with osteoporosis at high risk for fracture from the ATOM study. These findings are broadly consistent with results in postmenopausal women at high risk for fracture from the ACTIVE study and add to the growing body of evidence on how abaloparatide improves bone structure at the hip.

## Supplementary Material

Supplementary_materials_ziaf098

## Data Availability

Data that underlie the results reported in a published article may be requested for further research 6 months after completion of FDA or EMA regulatory review of a marketing application (if applicable) or 18 months after trial completion (whichever is latest). Radius will review requests individually to determine whether (i) the requests are legitimate and relevant and meet sound scientific research principles, and (ii) are within the scope of the participants’ informed consent. Prior to making data available, requestors will be required to agree in writing to certain obligations, including without limitation, compliance with applicable privacy and other laws and regulations. Proposals should be directed to info@radiuspharm.com.
