# Supplementary material for: The effect of abaloparatide on the proximal femur in men with osteoporosis assessed by three-dimensional dual-energy X-ray absorptiometry
Source: JBMR Plus. 2025 Jun 5;9(8):ziaf098. doi: 10.1093/jbmrpl/ziaf098 (PMC12278270; doi:10.1093/jbmrpl/ziaf098)
Supplement: Supplementary_materials_ziaf098 [file supplementary_materials_ziaf098.pdf]

Supplemental Table 1. **Mean Percent Change (95% CI) in DXA and 3D-DXA Parameters of Hip Subregions From Baseline to 12 Months**

	Abaloparatide (N=115)	Placebo (N=65)	LSM (95% CI) difference from PBO	*P* value vs PBO
**Cortical vBMD**				
Femoral neck	1.00 (0.38, 1.63)	−0.29 (−1.06, 0.47)	1.55 (0.56, 2.55)	0.0024
Trochanter	0.69 (−0.05, 1.43)	0.19 (−0.84, 1.23)	0.89 (−0.36, 2.13)	0.1632
Shaft	0.58 (−0.13, 1.30)	−0.08 (−1.00, 0.84)	0.99 (−0.19, 2.17)	0.0996
Total	0.54 (0.01, 1.07)	−0.03 (−0.69, 0.64)	0.75 (−0.12, 1.62)	0.0925
**Cortical sBMD**				
Femoral neck	2.50 (1.50, 3.50)	−0.43 (−1.42, 0.55)	3.15 (1.64, 4.66)	<0.0001
Intertrochanter	1.79 (1.02, 2.56)	0.01 (−1.01, 1.03)	2.17 (0.93, 3.41)	0.007
Shaft	1.77 (0.93, 2.61)	−0.05 (−1.08, 0.98)	2.16 (0.79, 3.52)	0.0021
Total	1.65 (0.93, 2.38)	0.15 (−0.75, 1.05)	1.87 (0.70, 3.04)	0.0020
**Cortical thickness**				
Femoral neck	1.70 (0.84, 2.57)	−0.21 (−1.31, 0.089)	1.83 (0.41, 3.25)	0.0120
Intertrochanter	1.10 (0.53, 1.68)	0.15 (−0.69, 0.99)	1.00 (0.03, 1.96)	0.0424
Shaft	1.13 (0.53, 1.74)	0.07 (−0.62, 0.76)	1.19 (0.23, 2.15)	0.0153
Total	1.10 (0.65, 1.56)	0.18 (−0.44, 0.81)	1.14 (0.38, 1.89)	0.0033

**Trabecular vBMD**				
Femoral neck	9.84 (6.87, 12.82)	−0.58 (−2.99, 1.82)	11.30 (7.01, 15.59)	<0.0001
Trochanter	6.47 (4.78, 8.15)	0.55 (−1.58, 2.67)	6.37 (3.58, 9.17)	<0.0001
Shaft	11.22 (8.30, 14.14)	−0.05 (−3.19, 3.10)	12.34 (7.80, 16.89)	<0.0001
Total	7.01 (5.16, 8.85)	−0.42 (−2.21, 1.37)	8.06 (5.24, 10.87)	<0.0001
**Integral vBMD**				
Femoral neck	5.35 (3.94, 6.75)	−0.61 (−1.83, 0.61)	6.23 (4.14, 8.32)	<0.0001
Trochanter	3.84 (2.86, 4.81)	0.37 (−0.97, 1.71)	3.70 (2.02, 5.38)	<0.0001
Shaft	4.19 (3.25, 5.14)	−0.04 (−1.08, 1.01)	4.58 (3.09, 6.08)	<0.0001
Total	3.67 (2.88, 4.47)	−0.10 (−0.88, 0.68)	4.00 (2.77, 5.22)	<0.0001
**aBMD**				
Femoral neck	3.68 (2.89, 4.48)	0.18 (−0.54, 0.91)	3.59 (2.41, 4.78)	<0.0001
Trochanter	2.24 (1.46, 3.02)	0.45 (−0.64, 1.55)	2.01 (0.70, 3.33)	0.0029
Shaft	2.50 (1.81, 3.19)	−0.36 (−1.05, 0.34)	3.16 (2.11, 4.22)	<0.0001
Total	2.50 (1.89, 3.11)	−0.10 (−0.77, 0.57)	2.83 (1.88, 3.78)	<0.0001

3D-DXA, three-dimensional dual-energy x-ray absorptiometry; ABL, abaloparatide; aBMD, areal BMD; BMD, bone mineral density; CI, confidence interval; LSM, least square mean; PBO, placebo; sBMD, surface BMD; vBMD, volumetric BMD.
